# A three step protocol for the development of an innovative footwear (shoe and sensor based insole) to prevent diabetic foot ulceration

**DOI:** 10.3389/fpubh.2023.1061383

**Published:** 2023-01-30

**Authors:** Liliana B. Sousa, Inês Almeida, Rafael A. Bernardes, Teófilo R. Leite, Rui Negrão, João Apóstolo, Anabela Salgueiro-Oliveira, Pedro Parreira

**Affiliations:** ^1^Health Sciences Research Unit, Nursing (UICISA: E), Nursing School of Coimbra (ESEnfC), Coimbra, Portugal; ^2^Indústrias e Comércio de Calçado S. A. (ICC), Sol-Pinheiro, Guimarães, Portugal

**Keywords:** diabetic foot ulcers (DFUs), prevention, footwear, shoes, sensor-based insoles

## Abstract

**Background:**

The incidence of diabetic foot ulceration (DFU) is increasing worldwide. Therapeutic footwear is usually recommended in clinical practice for preventing foot ulcers in persons with diabetes. The project Science DiabetICC Footwear aims to develop innovative footwear to prevent DFU, specifically a shoe and sensor-based insole, which will allow for monitoring pressure, temperature, and humidity parameters.

**Method:**

This study presents a three-step protocol for the development and evaluation of this therapeutic footwear, specifically: (i) a first observational study will specify the user requirements and contexts of use; (ii) after the design solutions were developed for shoe and insole, the semi-functional prototypes will be evaluated against the initial requirements; (iii) and a pre-clinical study protocol will enable the evaluation of the final functional prototype. The eligible diabetic participants will be involved in each stage of product development. The data will be collected using interviews, clinical evaluation of the foot, 3D foot parameters and plantar pressure evaluation. This three-step protocol was defined according to the national and international legal requirements, ISO norms for medical devices development, and was also reviewed and approved by the Ethics Committee of the Health Sciences Research Unit: Nursing (UICISA: E) of the Nursing School of Coimbra (ESEnfC).

**Results:**

The involvement of end-users (diabetic patients) will enable the definition of user requirements and contexts of use to develop design solutions for the footwear. Those design solutions will be prototyped and evaluated by end-users to achieve the final design for therapeutic footwear. The final functional prototype will be evaluated in pre-clinical studies to ensure that the footwear meets all the requirements to move forward to clinical studies.

**Discussion:**

The three-step study outlined in this protocol will provide the necessary insights during the product development, ensuring this new therapeutic footwear's main functional and ergonomic features for DFU prevention.

## 1. Introduction

Diabetes mellitus (DM) is considered one of the most impactful non-communicable diseases (NCD) affecting public health at a global level (1). In 2014, according to World Health Organization, almost 422 million adults worldwide were struggling with DM, following the trend registered in the last 36 years (2). In Europe, around 9.1% of the population presents DM diagnosis (3). In this context, the International Diabetes Foundation (4) estimates an increase in the diagnostics of DM by around 52% worldwide and 15% in Europe until 2045. According to the same document, in Portugal, between the age of 20 to 79 years old, DM has an approximate prevalence of 14.2%, which means a ratio of 1:7. According to Liu et al. (1), the worldwide increase in DM has been driven by global aging, economic growth, rapid urbanization, and nutritional transitions worldwide.

Although DM is a commonly referred disorder focused on by researchers and clinicians, the prevalence of undiagnosed cases is still high (5). The untreated DM threatens public health, accelerating comorbidity of micro and macrovascular complications, like neuropathies, retinopathies, deformities, and hampering future preventive measures (6). One of the most severe comorbidities is diabetic foot ulcers (DFU), which are usually associated with the loss of protecting sensibility, foot deformities, and the absence of foot pulses. The DFU is considered a common cause of amputation of inferior limbs (7). In fact, according to the same authors, DFU and amputations, which are consequences of diabetic neuropathy and/or peripheral arterial disease (PAD), are common and represent major causes of morbidity and mortality in people with DM. Early recognition and treatment of signs and symptoms in patients with DM, namely at the feet, is essential to delay or prevent these complications. It follows that appropriate therapeutic footwear and other wearable devices assume an indispensable role when prevention and treatment of DFU are clinical priorities (8–11).

Developing the devices mentioned above, with an application in a specific population with a particular need, receives clear contributions and necessary inputs from its end-user (the person with DM). Recent studies have proven that the efficacy of therapeutic interventions and devices can be improved with patients' and families' involvement in the whole process (12–14). In DM management and DFU prevention, footwear and related wearable devices, like insoles, can be considered medical devices with specific therapeutic or preventive goals. Intelligent devices and new technology has been highlighted to improve illness management and quality of life (15), mainly when patients are involved in their development (16, 17). In this sense, Human-Centred Design (HCD) is one of the most used methods that involve the user in the development process, according to the international directives (18, 19). Those directives were adapted in 2007 in Europe regarding the harmonized human factors and ergonomics standards for the analysis, design, verification and validation of safety-related usability through the medical device development cycle (20). The HCD model can be implemented to define, and design devices based on specific functional requirements and end users' needs (21). This method can predict potential usability errors due to ergonomic features, ensuring essential parameters related to human factors. In conclusion, this model improves safety, satisfaction, effectiveness and efficiency while reducing product recalls and modifications (22–25). In the last few years, the Technology Readiness Level (TRL) was reviewed to consider the technology's readiness for human support, human performance, ease of use, and user satisfaction (26). The Human Readiness Level (HRL) is used as a counterpart of the TRL to identify the level of readiness or maturity of a given technology concerning the use by intended users in the intended operational environment (26, 27). According to the HRL, different types of user research can be used during product development to identify improvement opportunities and minimize human error from the early stages of the design (26, 27).

The project *Science DiabetICC Footwear* aims to develop innovative, customized, and affordable footwear for DFU prevention. This product will have distinctive critical characteristics, such as new materials, better impact absorption, shape adjustment, minor abrasion, and greater recovery after loading. Also, the insole will incorporate sensors for assessing and monitoring essential parameters such as plantar pressure, temperature, and humidity. In this paper, considering HCD principles, we will describe a three-step protocol that will be used to accomplish the initial stages of TRL (levels 1 to 6) and HRL (basic research and development phase and technology demonstration phase) in this device development.

## 2. Methods

### 2.1. Study design

This study protocol was developed under the SPIRIT 2013 Guidelines (28), with some adjustments to pre-clinical trials in healthcare simulation research specificities (29, 30). The study employed a three-step protocol following the EU directives (31) implemented in Portugal by the Portuguese National Authority of Medicines and Health Products (INFARMED) (32). According to these recommendations and international standards in this field (18, 19), alongside with the TRL and HRL foundations, the HCD iterative method will be used, ensuring the involvement of the end-users throughout the development process to enhance the device's effectiveness, efficiency, and satisfaction.

In this sense, a three-step protocol supported by a mix-method design of qualitative and quantitative studies was developed and included:

(i) A first observational study will be used to specify the user requirements and contexts of use. Scientific research and preliminary development will occur on paper and in the laboratory (HRL 1 and 2).
(ii) The design solutions for shoe and insole, as well as semi-functional prototypes, will be developed and evaluated against initial requirements. It is expected to achieve a validated proof of concept that addresses human needs, capabilities, limitations, and characteristics (HRL 3 and 4).
(iii) A pre-clinical study protocol will evaluate the final functional prototype of the device (shoe and insole), ensuring that the device meets all the requirements to move forward to clinical studies in real contexts. This phase will demonstrate the fidelity levels of the device in laboratory environments (HRL 5 and 6).

### 2.2. Participants' recruitment and sample size

In the study's first phase, a random sample will be extracted from the available database of 919 diabetic patients from a selected primary care organization in Portugal. Slovin's formula is used to calculate the sample size (*n*) given the population size (*N*):


(1) $$\begin{array}{l} n\;=\;\frac{N}{1+Ne^{2}} \end{array}$$

where, *n* = number of samples, *N* = total population, and e = margin of error. This formula applies when estimating a population proportion using a confidence coefficient of 95% (33). A random extraction of the 279 diabetic patients will be done from the original database using the Random between function in Microsoft excel. A local family health nurse will contact each selected participant to schedule the assessment according to the eligibility criteria described in Table 1.

**Table 1:** Eligibility criteria for participants.

	**I phase**	**II phase**	**III phase**
**Inclusion**			
• Age equal or superior to 18	X	X	X
• Able to communicate in Portuguese	X	X	X
• Collaborative and oriented	X	X	X
• With a diagnosis of Diabetes type 1 or 2	X	X	X
• Informed consent to participate in the study	X	X	X
**Exclusion**			
• Inability to walk freely without any assistive device	X		X
• Active foot ulcers at the time of assessment			X
• Any type of lower limb amputation			X
• Major foot deformities (e.g., cavus or flat foot, Charcot)			X
• Vascular disease, claudication, retinopathy, nephropathy, and any orthopedic (e.g., fracture) or neurological (e.g., stroke) impairment that could influence gait			X

I. Observational study (user requirements and contexts of use); II. Design solutions evaluation (semi-functional prototypes); III. Pre-clinical study (final functional prototypes).

Regarding sample size for the usability tests (second and third phases), around 15–25 participants are usually considered to enroll, 15 being the acceptable minimum number according to the regulatory entities of the USA (34). Although these parameters in the EU are not well-established, the U.S. Food and Drug Administration's (FDA) orientation guidelines for medical devices highlight the need to balance the samples' heterogeneity and homogeneity, reflecting the target population as much as possible. According to the standard AAMI/IEC/TIR 62366-2 (19):


$$\begin{array}{l} R\;=\;1-(1-P{)}^{n} \end{array}$$

where, *R* = cumulative probability of detecting a usability problem; *P* = probability of a single test showing a usability problem; *n* = number of participants. According to this, there are residual returns on detecting usability problems when the sample size exceeds 10 for each distinct user group. These participants will be randomly extracted from the database of the participants included in the first phase of the study.

### 2.3. Materials and equipment

All the procedures in observational and pre-clinical tests will be conducted in a laboratory setting with three main areas previously prepared (i) clinical evaluation of the foot, (ii) evaluation of the participants' shoes, plantar pressure and foot 3D analysis, and (iii) quality of life assessment and interviews. The CRF will include several sections necessary to accomplish the main purposes of each study phase defined in each protocol.

Some instruments and stratification checklists will be used along the three-step study, namely: (i) Portuguese version of the Graffar scale (35) composed by seven items, in order to stratify the socioeconomic level of the participants; (ii) the Portuguese (36) and international (37) classifications will be used to determine the risk level for the development of DFU, both reported by the researcher according to variables like neuropathy, ischaemia, foot deformities, ulcer previous history, amputation, loss of protective sensitivity and peripheral arterial disease; (iii) a questionnaire will be used to evaluate the footwear, which will require a ruler, a tape measure and a scale (38); (iv) EUROHIS QoL 8 (39) will be used to estimate the quality of life of the participants; (v) usability questionnaire with 42 items in a 7-point Likert scale to assess the usefulness, ease of use, ease of learning, satisfaction and intention to use (40).

Specific instruments and materials will be used for the clinical evaluation of the foot, namely: (i) monofilament 10 g of Semmes-Weinstein (41) will be used to assess the sensitivity loss (42); (ii) the 128 Hz tuning fork (43) will be used to assess the vibratory sensitivity; (iii) cotton will be used in the plantar region for the tactile sensation; (iv) the pinprick test will be used to assess the discrimination sensitivity; (v) the reflexion hammer will be used to evaluate the aquilian and rotulian tendons reflexes; (vi) the handheld Doppler device will be used to determine the Ankle-brachial Pressure Index (ABPI) values.

The pedobarographic measurements will be performed with either (i) a platform-based system (EMED; Novel GmbH, Munich, Germany) for dynamic barefoot plantar pressure assessment, with a sampling frequency of 50 Hz and resolution of 2 sensors/cm2 for a network of 2736 sensors; and (ii) an insole-based system (PEDAR X; Novel GmbH, Munich, Germany) for in-shoe plantar pressure evaluation, that comprises flexible 2 mm thick insoles with a matrix of 99 capacitance-based sensors each sampling at 50 Hz placed in the shoes. According to Putti et al. (44), the PEDAR X is one of the most commonly used systems for in-shoe pressure measurement, traducing good repeatability and consistency of measurements. Also, the EMED system is among humans' most frequently used clinical tools for barefoot pressure measurement worldwide (45). A 3D scanner (Feetbox 3D; Sidas and Corpus.e) will also be used to determine foot volumetric parameters.

### 2.4. Outcomes

The main purpose of the first phase will be to explore the concept of the new device for DFU prevention. According to this, the main outcomes will be the definition of the contexts of use, users' needs and requirements, and the footwear design features (Table 2). In phase II, the main outcome will be the definition of the design features of the device and the development of the semi-functional prototypes. Additionally, the main outcomes regarding the evaluation of those prototypes will be related to ergonomic and functional aspects of the footwear, as well as to users' satisfaction and intention to use. These outcomes will also be evaluated in the last phase regarding the functional prototype. Furthermore, the usability testing with the functional prototype will enable the assessment of safety, efficiency and/or effectiveness outcomes (regarding the decreased plantar pressure in critical areas of the foot), ensuring that the medical device accomplishes the needed legal requirements before testing in real settings.

**Table 2:** Outcomes in each phase of the study.

	**I phase**	**II phase**	**III phase**
Contexts of use	X		
User needs and requirements	X		
Design	X	X	X
Ergonomic and functional features		X	X
Satisfaction and intention to use		X	X
Safety			X
Efficiency and/or effectiveness			X

I. Observational study (user requirements and contexts of use); II. Design solutions evaluation (semi-functional prototypes); III. Pre-clinical study (final functional prototypes).

### 2.5. Procedures

Table 3 explains the interventions in each phase of the study, according to the main purposes defined. All the procedures will be explained to the participants in the three phases, and the consent form will be signed. After the informed consent, data collection will include sociodemographic (e.g., age, gender, education) and clinical variables (depression, physical impairment, smoking or alcohol habits, height, weight, body mass index, prior history of ulceration, angioplasty or vascular surgery), considering important predictive risk factors for diabetic foot ulceration (42, 46, 47). A clinical foot evaluation (phases I and III) will be performed by members of the research team and should include a visual inspection of the foot (skin inspection, foot deformities), vascular (peripheral arterial disease; PAD) and neurological (neuropathy) assessment. Also, in the first phase, a footwear assessment will be performed (as well in phase III), followed by a 3D foot scan. The plantar pressure assessment (in-shoe and barefoot) will be performed in all three phases. The studies will be finished by evaluating the quality of life (phases I and III) and an interview (in all phases, with specific purposes detailed above). Specifically, in phase III, the functional prototypes will be evaluated by their end-users (individuals with diabetes) thorough usability testing. The specific procedures for visual inspection, vascular and neurological evaluation, footwear assessment, 3D scan and plantar pressure evaluation are presented below.

**Table 3:** Interventions in each phase of the study.

	**I phase**	**II phase**	**III phase**
Demographic data	X	X	X
Clinical data	X	X	X
Clinical evaluation of the foot	X		X
Footwear evaluation	X		X
Plantar pressure assessment	X	X	X
3D foot assessment	X		
Quality of life assessment	X		X
Interview	X	X	X
Usability testing		X	X

I. Observational study (user requirements and contexts of use); II. Design solutions evaluation (semi-functional prototypes); III. Pre-clinical study (final functional prototypes).

#### 2.5.1. Clinical evaluation of the foot

The foot visual inspection will enable the evaluation of previous lower limb amputations (42, 46), as well as foot deformities (48) that contribute to ulcer development (49). Also, several articular, ungueal and tegumentary deformities will be evaluated, such as erythema, callus formation, deformity, skin integrity, and fungal infections of skin and nails (48).

For vascular assessment, along with pedal pulse palpation, the ABPI is a widely utilized test for diagnosing PAD (50), whose principle is to compare the blood pressure in the lower extremities to central blood pressure. The ABPI will be calculated by measuring the systolic blood pressure with a Doppler on both arms (at the brachial artery) and legs (at posterior tibial and dorsalis pedis arteries), and the higher value is taken for application in the following formula:


$$\begin{array}{l} ABPI\;=\;\frac{higher\;value\;(posterior\;tibial\;or\;dorsalis\;pedis)}{higher\;value\;\left(brachial\right)} \end{array}$$

ABPI values are considered: (i) normal between 1.0 and 1.4; (ii) borderline PAD with values between 0.91 and 0.99; (iii) PAD in values below 0.9; (iv) severe PAD in values below 0.4; (v) values above 1.4 suggest calcified rigid and non-compressible arterial walls (51). Also, the temperature will be measured by an infrared thermometer [according to the Houghton et al. conclusions (52)] in both feet as well as overall body temperature, enabling the comparison between the foot and overall temperature, but also the comparison of temperature in the same contralateral anatomical foot regions (14, 52, 53).

The neurological assessment will involve the detection of sensory loss through the 10g monofilament, vibration perception having a tuning fork of 128 Hz, pain sensitivity through the pinprick, kinaesthetic evaluation by cotton, along with the neuropathic symptoms and tendon reflexes (aquilian and rotulian) assessment (42, 54). Regarding sensitive evaluation, the most commonly used is the Semmes-Weinstein 10 g monofilament, calibrated as it requires 10 g of force for bending on touching the foot skin. After applying the filament to the patient's hands to demonstrate what the sensation feels like, the filament will be used in five different regions on each foot (hallux, first metatarsal, third metatarsal, fifth metatarsal, and heel), ensuring that the participant cannot see whether or where the examiner applies the filament: apply the filament perpendicular to the skin surface with sufficient force to cause the filament to bend or buckle for nearly 2 s. In each area, the test is repeated three times, with one “mock” application in which the filament is not applied. A protective sensation is present at each site if the patient correctly answers two out of three applications.

After applying the tuning fork to the patient's hands to demonstrate the sensation, the 128 Hz tuning fork test will be used to determine vibration sensation. The participant will be requested to report the vibration perception: apply the tuning fork perpendicularly and with constant pressure at the first toe' dorsum to the nail bed's proximity to the bone prominence. On each toe, the test will be repeated three times, with one “mock” application in which the tuning fork is not vibrating, ensuring that the participant cannot see whether or where the examiner applies the tuning fork. The test is positive if the patient correctly answers at least two out of three applications.

The pinprick-pain stimulus will be performed on the patient's hands first and then twice at the first toe' head (right and left lower limb), ensuring the participant cannot see whether the examiner uses a sharp or dull surface. This test was used for the perception of sharp touch with a toothpick. The patient's pain sensitivity was obtained using this instrument in some regions of the foot (43). The cotton will be used in the right and left lower limbs' plantar area three times, with a “mock” application, to assess the sensory neuropathy detection (42). In both cases, the participants will be asked if they feel the sharp or dull surface of the pinprick and the cotton on the plantar surface.

#### 2.5.2. Risk assessment

To categorize the grade of risk, all the data previously collected will be analyzed to determine the current ulcer risk according to the international (37) and national (36) classification systems (Table 4).

**Table 4:** Risk assessment classifications.

	**Category**	**Ulcer risk**	**Characteristics**
IWGDF (2019)	0	Very low	No LOPS and No PAD
	1	Low	LOPS or PAD
	2	Moderate	LOPS + PAD, or LOPS + Foot deformity, or PAD + Foot deformity
	3	High	LOPS or PAD, and one or more of the following: - history of a foot ulcer - a lower-extremity amputation (minor or major) - end-stage renal disease
DGS (2010)		Low	Absence of risk factors
		Moderate	Presence of neuropathy
		High	Presence of ischemia and/or neuropathy and/or foot deformity, or, history of a healed foot ulcer, or, previous amputation

LOPS, Loss of protective sensation; PAD, Peripheral artery disease.

#### 2.5.3. Footwear assessment

To evaluate the footwear characteristics, a specific tool developed by Barton et al. (38) will be applied, which covers the following items: (i) fit of the shoe (length, width, depth); (ii) general features (age of shoe, footwear style, upper and outsole materials, weight, length, weight/length); (iii) general structure (heel and forefoot height, longitudinal profile, last, fixation, forefoot sole flexion point); (iv) motion control properties (density, fixation, heel counter stiffness, midfoot sole sagittal and frontal stability); (v) cushioning (presence, hardness at lateral midsole, medial midsole, and heel sole); (vi) wear patterns (presence, midsole, tread pattern, outsole wear pattern).

#### 2.5.4. Plantar pressure assessment

A quantitative assessment of dynamic foot plantar pressures will be performed, including in-shoe and barefoot examination (55–57). In the barefoot plantar pressure procedure, the participants will be instructed to walk barefoot over the foam runway, with the EMED platform located in the middle of the runway. Participants will be instructed to walk at an average pace and ensure that a minimum of three steps are taken before and after contacting the platform. This three-step protocol may offer consistent results and avoid unnecessary foot loading, especially in individuals with diabetic peripheral neuropathy (56). At least three to five (55) assessments will be required to ensure the reliable evaluation of pressures. Before starting the recording, all participants will be allowed a familiarization period consisting of two practice trials.

For the insole-based PEDAR system, participants will be fitted with the correct insoles for their shoe size (ensuring that the insole will cover the entire plantar surface) and will use their usual footwear. The assessment procedure involves the need to wear a waist belt containing a battery and a wireless Bluetooth, allowing real-time connection and data storage to a laptop computer. The participants will be instructed to walk a distance of ~15 m before turning and returning to the place where they started. Insole pressure data will be collected during both of these walks, ensuring data for at least 12 steps from each participant, which is the minimum number of steps required to obtain reliable in-shoe pressure data in individuals with peripheral diabetic neuropathy (58). Also, before pressure assessment, a zero-calibration will be performed by unloading each measurement insole, and all participants will be allowed two practice trials before recording.

#### 2.5.5. 3D foot assessment

A 3D scanner (LAVORO) will be used to determine participants' foot volumetric parameters. The main parameters being evaluated are foot size (EU size), foot length in centimeters (cm), width (cm), ball girth (cm), instep height (cm), heel with (cm), girth calf 15 (cm), girth calf 25 (cm), girth angle floor and gait angle ankle, both in degrees. Participants will be asked to wear a pair of sterilized socks with specific sensors compatible with the platform. Afterwards, they'll assume a static position on the platform's top, with their feet equally distanced and parallel to their shoulders. The platform will smoothly scan the previously mentioned variables, which will be stored in a local computer.

#### 2.5.6. Quality of life assessment

The EUROHIS QoL8 (39) will be used for the quality of life assessment. This is an 8-item index that was developed as an adaptation of the WHOQOL-100 and WHOQOL-Bref (the WHO cross-cultural and generic instruments to assess the quality of life). The EUROHIS QoL8 revealed good internal consistencies across a range of countries. It showed acceptable convergent validity with physical and mental health measures, discriminating well between healthy individuals and those with longstanding conditions such as diabetes (59).

#### 2.5.7. Interview

To complete the study, the participants will be asked to participate in an interview with specific purposes according to the phase of the study. In the first phase (observational study), the interview will characterize the functional and ergonomics aspects of their everyday footwear and identify their difficulties, needs, wellbeing and potential limitations in daily activities related to foot condition and footwear. Also, the interview will identify personal preferences regarding essential characteristics of the footwear that will be developed, such as style, color, fastening system, shape, or materials.

The interview in phase II (regarding the design solutions and semi-functional prototypes) will enable an assessment of the design solutions and determine any necessary modifications to improve the footwear and/or the insole. In phase III, the functional prototype will be evaluated alongside specific usability tests (described above), and an interview will determine potential suggestions for the device improvement.

#### 2.5.8. Usability testing

Usability is “the extent to which a user can use a product to achieve goals with effectiveness, efficiency, and satisfaction in a specific context” (18). Phase II will use the design solutions and semi-functional prototypes in order to evaluate if they meet the initial requirements. Phase III, with the final functional prototype, will ensure that the device meets all the requirements to proceed with clinical studies. In both phase II (semi-functional prototypes) and phase III (functional prototype), along with the interviews, the Usability Questionnaire (40) will be used for the functional prototype evaluation. Specific usability testing will be implemented using the footwear prototype (shoe and insole) in laboratory settings, following the same protocol described for the PEDAR system. The same participant will perform the plantar pressure assessment barefoot, in-shoe (with regular footwear), and with the prototype of the footwear to compare the plantar pressure profiles.

### 2.6. Data collection, management, and analysis

Study participants' identification numbers (ID) will be used, and all data will be anonymized for subsequent analysis and reports/publications. Individual information to be collected includes demographic (gender, age), academic qualifications (degree), and professional data (clinical experience, work setting) of the nurses eligible to perform the usability tests. The names of the participants on the consent forms will be stored separately in locked cabinets accessible only by named personnel.

Statistical analysis of the collected data will be performed using the Statistical Package for the Social Sciences, version 24 (IBM SPSS Statistics 24; SPSS Inc., Chicago, IL, USA). Means, standard deviations, frequencies, and percentages will be used as descriptive statistics (or median values and interquartile ranges for skewed data). The outcomes in the two groups in each study phase will be examined to detect the effect of group allocation through inferential statistics (Student's *t*-test for independent and paired samples, or non-parametric equivalents, Mann-Whitney U and Wilcoxon tests; X2 test or Fisher's exact test), considering a statistical significance level of 0.05 (two-sided significance level of 5%). For qualitative analysis (interviews), the content analysis technique will be conducted (60) after the transcription of the individual interviews.

### 2.7. Ethical considerations

The study protocol was reviewed and approved by the Ethics Committee of the Health Sciences Research Unit: Nursing (UICISA: E) of the Nursing School of Coimbra (ESEnfC; Number P631/10-2019) and by the Ethics Committee of the Health Regional Administration (ACeS; Number 70/2020). The pre-clinical stages were defined according to the legal requirements of the European Union (31) and the ISO norms related to ergonomics and usability assessment of medical devices (18, 19, 61–63).

The eligible participants will receive written and oral information about the study. Written informed consent and a non-disclosure agreement (NDA) will be requested. Participants will be assigned an ID number to maintain anonymity and be easier to conciliate all the collated data, which will be used in all data collection instruments (case report form; software for plantar pressure and foot 3D analysis). Personal information will be separated from the main data collection instruments and will not be shared. All the documentation related to the study will be saved in locked cabinets only accessible by the study members. In the same way, the data collected by plantar pressure and 3D software will be obtained and kept in a project computer only accessible by study team members. All collected data will be exclusively for this study, and the confidentiality of participants will always be maintained.

### 2.8. Dissemination

Due to the absence of specific guidelines for pre-clinical studies with medical devices, upon completion of the several tests in the three phases, the data obtained will be reported with the necessary adjustments for health care simulation research specificities (29, 30). The data will not be publicly available but accessible from the principal investigator on reasonable request. The research results will be disseminated to open-access, peer-reviewed journals and national and international scientific meetings. Authorship will be considered according to the recommendations of the International Committee of Medical Journal Editors (64) regarding the contributions to the design, conduct, interpretation, and reporting of the pre-clinical data (65).

## 3. Discussion/conclusion

The development of medical devices has increased over the last years, playing an important role in clinical practice, not only by improving care practices but also by directly influencing patients' wellbeing and quality of life.

The development of a medical device should be an iterative process, where de HCD model plays an essential role in the several stages of product development, according to the international directives that were adapted in Europe since 2007 (18, 19). The involvement of the end-users in product development ensures that the device meets the users' needs and preferences, increases device safety, effectiveness and efficiency, reducing product recalls and modifications (22–25).

This study protocol was developed to provide specifications regarding the end users' involvement in developing footwear and insole to prevent DFU since the definition of users' requirements and contexts of use and evaluation of design solutions and prototypes. Scientific research and preliminary development will be made to verify the clinical need of the end-users (health professionals and diabetic patients), as well as the review the existing medical devices and procedures used to treat or prevent the condition (66). The end-users input regarding their needs and requirements will enable the definition of early design inputs for the development of the initial prototypes. The pre-clinical studies will ensure that the device successfully meets the user needs and requirements, along with the first inputs regarding device validation (feasibility studies), safety and user satisfaction (66).

A detailed description of the activities that will be carried on in each phase was conducted, and effectively will assist both industrial and technological partners in product development and should result in successful and high-quality products.

## Data availability statement

The original contributions presented in the study are included in the article. Further inquiries can be directed to the corresponding author.

## Author contributions

Conceptualization: LBS and PP. Writing original draft: LBS and IA. Writing review and editing: RAB, RN, AS-O, and PP. Supervision: PP, JA, and TRL. Funding: PP and TRL. All authors have read and agreed to the published version of the manuscript.
